# Common Single Nucleotide Polymorphism of *TMPRSS6*, an Iron Regulation Gene, Associated with Variable Red Blood Cell Indices in Deletional α-Globin Genotypes

**DOI:** 10.3390/genes13091502

**Published:** 2022-08-23

**Authors:** Thidarat Suksangpleng, Waraporn Glomglao, Vip Viprakasit

**Affiliations:** 1 Siriraj-Thalassemia Center, Faculty of Medicine Siriraj Hospital, Mahidol University, Bangkok 10700, Thailand; 2 Division of Hematology/Oncology, Department of Paediatrics, Faculty of Medicine Siriraj Hospital, Mahidol University, Bangkok 10700, Thailand

**Keywords:** iron, red blood cell indices, *TMPRSS6*, thalassaemia

## Abstract

Red blood cell (RBC) indices, including mean corpuscular volume (MCV) and mean corpuscular haemoglobin (MCH), have been widely used for primary screening for thalassaemia (thal) syndromes. Recently, a single nucleotide polymorphism (SNP) rs855791 of *TMPRSS6*, an iron regulation gene involved in the substitution of a nucleotide between thymine (T) and cytosine (C) in exon 17 resulted in an amino acid change, p.Val736Ala (V736A), has been described to associate with RBC indices. The objective was to study the effects of common SNP V736A on RBC indices in deletional α-thal variations. SNP rs855791 genotypes were identified from 433 Thai volunteers, including 32.6% males and 67.4% females with an average age of 23.0 ± 8.7 years. These populations included individuals (82.4%) who had normal globin genotype (αα/αα, ββ) and α-thal carriers, which were divided into two subgroups, including α^+^-thal (-α/αα) (14.1%) and α^o^-thal (--/αα) (3.5%). Among three SNP genotypes, the C allele gradually expressed higher MCV and MCH than those of the T allele in both α^+^- and α^o^-thal traits. Importantly, SNP rs855791 of *TMPRSS6* responded to α-globin deletions for sustaining RBC sizes and haemoglobinisation in α-thal carriers.

## 1. Introduction

Type II transmembrane serine protease 6 (matriptase 2) encoded from the transmembrane serine protease 6 gene (*TMPRSS6*) mapped on 22q12.3 in hepatocytes. *TMPRSS6* plays a role in iron homeostasis by regulating the hepcidin hormone. The negative regulation of hepcidin transcription by *TMPRSS6* results in promoting intestinal iron absorption to maintain iron homeostasis [1,2].

*TMPRSS6* has been described as associated with a type of iron deficiency anaemia (IDA), a condition termed iron-refractory iron deficiency anaemia (IRIDA), which presents highly variable haemoglobin (Hb) levels and mean corpuscular volume (MCV) resulting in microcytic hypochromic anaemia and the iron parameters presents a low transferrin saturation, whereas serum ferritin level is normal and hepcidin is high [3,4,5]. A variety of *TMPRSS6* defects, including homozygous or compound heterozygous variants, was described previously in the IRIDA patients. The *TMPRSS6* variants resulting in the IRIDA include the frameshift variants such as c.497delT; p.Leu166Argfs*37 and c.1904_1905dup; p.Lys636Alafs*17 and the missense variants such as c.2383G>A; p.Val795Ile and c.2105G>T; p.Cys702Phe [6].

One common SNP variation, termed rs855791, has been described to involve iron status and red blood cell (RBC) indices, including MCV and mean corpuscular haemoglobin (MCH). The SNP rs855791 is a common variant of *TMPRSS6*playing a role in the determination of the various levels of RBC indices and iron parameters in the population [7,8,9]. The SNP locates in exon 17 of *TMPRSS6* as T>C substitution in nucleotide position 2207, inducing missense change from valine (V) to alanine (A) as GTC>GCC; V736A. The three SNP genotypes include homozygous C allele (C/C), T allele (T/T), and heterozygous T/C. Several studies of allele frequency and prevalence of SNP variation were described. The C allele frequency was highly distributed in the Caucasian population, similar to the Rwandan population in Sub-Saharan Africa [10]. On the other hand, different allele frequencies were reported in the Asian population, where T alleles, including T/T and T/C genotypes, presented at higher frequencies than those of the C/C genotype presented in the Taiwanese, Japanese, and Indian populations [9,10,11,12].

Interestingly, different T and C alleles showed effects on haematological parameters, RBC indices, and iron status in various populations. Higher levels of Hb concentration, RBC index parameters and iron parameters (serum iron, transferrin saturation) were obviously presented in the C allele [7,8,9,11,12]. These suggest that SNP variation of rs855791 should be a genetic factor to determine the haematological and RBC index parameters and influence inherited IDA conditions. For the Thai population, the allele frequency and effects of SNP variation on RBC indices and IDA conditions are still unclear because the Thai population gain genetic factor complexity involved in haematological parameters. The major genetic factor which influences various haematological parameters and RBC indices is α-globin gene copies, which encode the α-globin peptide chain incorporated into globin complex and heme to form a haemoglobin molecule in RBC. The deletions of α-globin gene copies induced different α-thalassemia (α-thal) genotypes, including single deletional α^+^-thal (-α/αα), double deletional α^o^-thal (--/αα), and homozygous single-deletion α^+^-thal (-α/-α) [13]. These deletional types of α-thal induced variable haematological parameters and RBC indices in the Thai population [14,15]. The study of the effects of SNP variation in *TMPRSS6* on RBC indices in the Thai population carrying different genotypes of α-globin deletion would be helpful for understanding the genetic factors that determine variable RBC characteristics and IRIDA conditions. Therefore, the aim of this study is to determine the effects of common SNP rs855791 in *TMPRSS6* on RBC indices and iron status in the Thai population carrying different genotypes of α-globin deletions.

## 2. Materials and Methods

### 2.1. Specimen Collection

This research project has been approved by the local ethical committees at Siriraj Internal Review Board of Faculty of Medicine Siriraj Hospital, Mahidol University and conducted in accordance with the Declaration of Helsinki. Healthy individuals, including 141 men and 292 women aged between 15–40 years, were recruited from the Thalassemia Screening Project in Ao Udom, Laem Chabang City Municipality, Chonburi, Thailand, from November 2012 to February 2015. Individuals with a medical history of haemophilia, IDA, myelodysplastic syndrome, menorrhagia, and sepsis were excluded. The 6-mL ethylenediamine tetraacetic acid (EDTA) whole blood samples and 6-mL clotted blood were collected.

### 2.2. Routine Haematological Workup

All EDTA-whole blood samples were analysed; the haematological parameters, included red blood cell count (RBC), Hb, haematocrit (Hct), red cell distribution width (RDW), and RBC indices, including MCV, MCH, and mean corpuscular haemoglobin concentration (MCHC) using COULTER^®^ Ac·T™ 5diff OV (Beckman Coulter, Miami, FL, USA). Hb profiles were identified based on high-pressure liquid chromatography (HPLC) by using VARIANT^TM^ II Hemoglobin Testing System (Bio-Rad, Hercules, CA, USA).

### 2.3. Analysis of α-Globin Mutations

The genomic DNA was extracted from an EDTA-whole blood sample using QIAamp^®^ 96 DNA Blood Kit (QIAGEN, Hilden, Germany). The polymerase chain reaction (PCR) based molecular analysis was used to analyse the α-globin gene genotypes, including deletional and non-deletional mutation [16,17,18].

### 2.4. Analysis of TMPRSS6 SNP rs855791 Genotypes

Genotypes of *TMPRSS6* SNP rs855791 were analysed from each genomic DNA sample with a PCR-restriction fragment length polymorphism (PCR-RFLP) technique modified from a previous study [9]. The specific PCR fragment containing SNP rs855791 in exon 17 of *TMPRSS6* was amplified by using the specific primers MT2-17F 5′-GTGGGCAGAGCAGGAGAGAAG-3′and MT2-17R 5′-GATGTGAGCAAAGGGCCAGAC-3′. The PCR cycle was performed by initial denaturing at 94 °C for 5 min followed by 34 cycles, including denaturing at 94 °C for 1 min, annealing at 62 °C for 1 min, and extension at 72 °C for 1 min and final extension at 72 °C for 5 min by using BIO-RAD T100™ Thermal Cycler (BIO-RAD, Hercules, CA, USA) followed by analysis PCR product by 1.5% agarose gel electrophoresis. For RFLP, the PCR products were digested with 5 units of *Stu*I restriction enzyme (New England BioLabs, Ipswich, MA, USA), followed by incubation at 37 °C for 4 h. The digested fragments were analysed by 3% agarose gel electrophoresis.

### 2.5. Determination of Serum Ferritin

The serum ferritin level was measured from serum collected from a 6-mL clotted blood sample using Elecsys Ferritin Assay commercial kit (Roche Diagnostic GMBH, Penberg, Germany) [19].

### 2.6. Statistics Analysis

All data of RBC indices, globin gene mutation, iron parameters, and allele frequencies of *TMPRSS6* SNP rs855791 were determined by statistical analysis using IBM®SPSS®Statistics 18 (SPSS, Chicago, IL, USA). The independent *t*-test was used to analyse the association between genotypes of *TMPRSS6* SNP rs855791 and the RBC index parameters. In each group of α-globin genotypes, data of RBC indices (MCV and MCH) of different SNP genotypes (C/C, T/C and T/T) were calculated for significant differences by independent *t*-test at *p* ≤ 0.05.

## 3. Results

### 3.1. Studied Population

Total 433 cases (age: 23.0 ± 8.7 years old, male: N = 141 (32.6%), female: N = 292 (67.4%)) of normal β-globin gene (ββ) included 3 genotypes of α-globin gene; normal (αα/αα) (N = 357, 82.4%), -α/αα (N = 61, 14.1%), and --/αα (N = 15, 3.5%) (Table 1).

### 3.2. RBC Indices Characteristics

**Figure 1:**
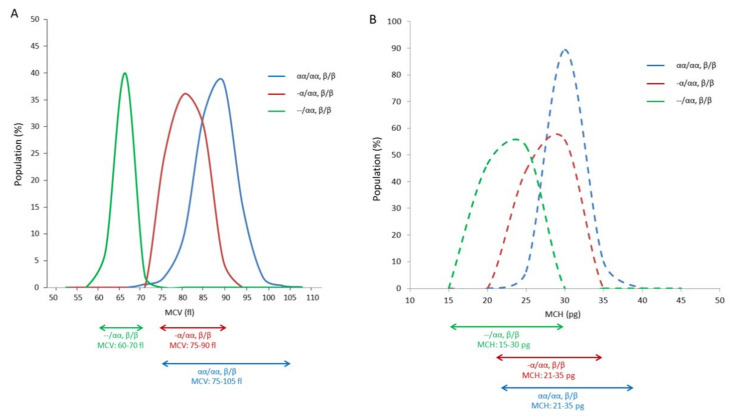
Ranges of RBC index parameters: MCV (**A**) and MCH (**B**) among the population of normal globin gene (αα/αα, β/β), α^+^-thal (-α/αα, β/β), and α^o^-thal (--/αα, β/β).

RBC indices: MCV and MCH were analysed by their ranges in α-globin genotypes: normal α-globin alleles (αα/αα), α^+^-thal (-α/αα), and α^o^-thal (--/αα). The MCV and MCH ranges were distinguished between normal α-globin alleles and α^o^-thal and between α^+^-thal and α^o^-thal, whereas normal α-globin alleles and α^+^-thal provided overlapping ranges of MCV and MCH that could not identify these two genotypes, as shown in Figure 1.

### 3.3. Genotypes of TMPRSS6 SNP rs855791

The genotypes of *TMPRSS6* SNP rs855791 on exon 17 (GTC>GCC; V736A) were identified by PCR-RFLP and presented three genotypes, including homozygous C (C/C), homozygous T (T/T), and heterozygous T/C in each population of different α-globin genotypes. The allele frequency of SNP rs855791 between T and C alleles was presented as 0.6 to 0.4 in male and female populations, as shown in Table 2.

### 3.4. Variation of RBC Indices Associated with SNP rs855791 Genotypes

**Figure 2:**
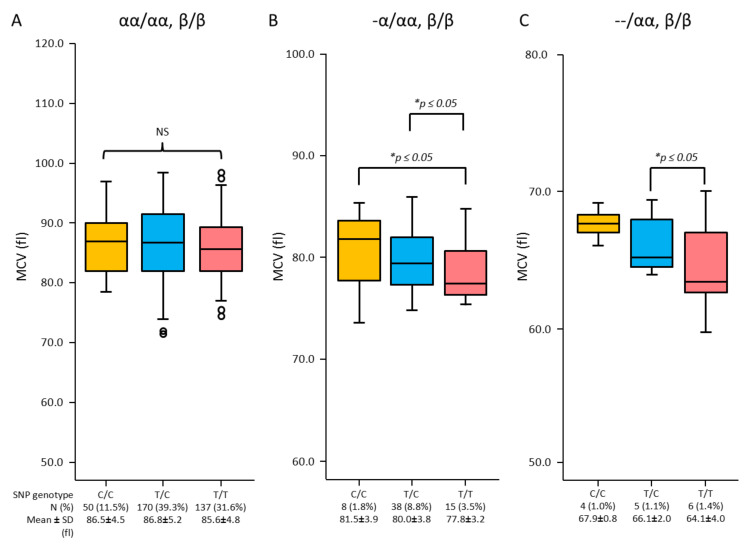
Characteristics of MCV among SNP rs855791 genotypes (C/C, T/C, T/T) of *TMPRSS6* in the population of normal α-globin gene (αα/αα) (**A**), α^+^-thal (-α/αα) (**B**) and α^o^-thal (--/αα) (**C**) (*significant difference at *p*-value ≤ 0.05 from independent *t*-test; NS, non-statistical significance at *p* > 0.05).

**Figure 3:**
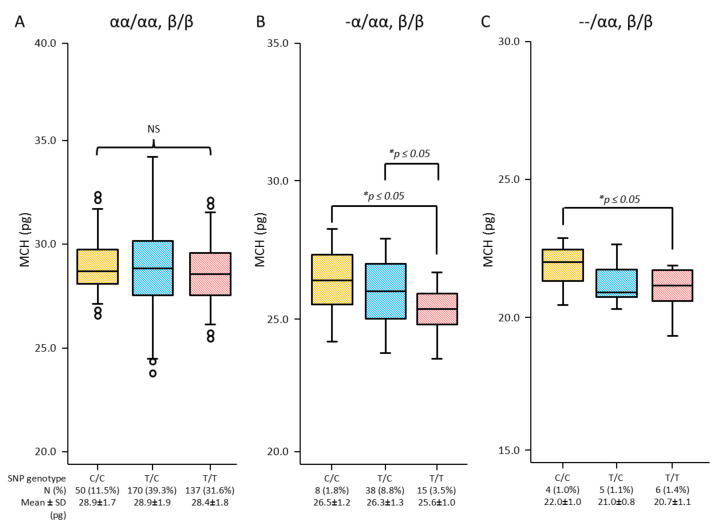
Characteristics of MCH among SNP rs855791 genotypes (C/C, T/C, T/T) of *TMPRSS6* in the population of normal α-globin gene (αα/αα) (**A**), α^+^-thal (-α/αα) (**B**) and α^o^-thal (--/αα) (**C**) (*significant difference at *p*-value ≤ 0.05 from independent *t*-test; NS, non-statistical significance at *p* > 0.05).

Results showed no effects of either the C or T allele on MCV and MCH in individuals with normal globin genes. However, the effects of SNP rs855791 genotypes on MCV and MCH were demonstrated in α-thal deletions. Increased MCV and MCH are gradually expressed by SNP genotype C/C, followed by T/C and T/T in α^+^- and α^o^-thal genotypes (Figure 2 and Figure 3).

### 3.5. Iron Status in SNP Variation of TMPRSS6

**Figure 4:**
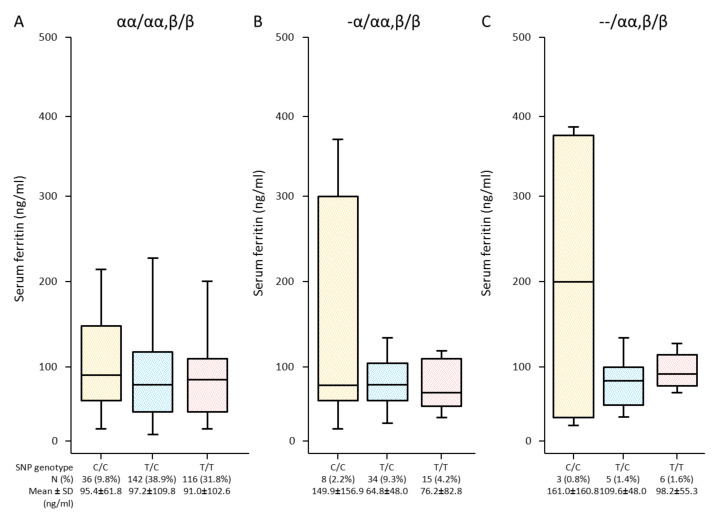
Association of serum ferritin levels with *TMPRSS6* SNP rs855791 variation (C/C, T/C, T/T) determined in three studied populations, including normal α-globin genotype (αα/αα) (**A**), α^+^-thal (-α/αα) (**B**), and α^o^-thal (--/αα) (**C**). (Significant difference at *p*-value ≤ 0.05 from independent *t*-test).

The effects of *TMPRSS6* SNP rs855791 on serum ferritin levels were also analysed in three populations of α-globin genotypes. The increasing fashions of serum ferritin levels were found with the C allele in both α^+^- and α^o^-thal genotypes but did not reach statistical significance (Figure 4).

## 4. Discussion

Several polymorphisms associate with iron homeostasis in human, including p.C282Y (c.845 G>A; rs1800562) and p.H63D (c.187 C>G; rs1799945) of human hemochromatosis protein (*HFE*), the SNP -8CG and -98GC of ferroportin (*FPN1*), SNP c.−582A>G; rs10421768 on the hepcidin anti-microbial peptide (*HAMP*) promoter region, p. P570S (C>T; rs1049296) of transferrin (*TF*) and p.V736A (T>C; rs855791) of *TMPRSS6* [7,20,21,22,23,24]. *TMPRSS6* is a type of trans-membrane serine protease mainly involved in iron homeostasis resulting in resistance to IRIDA disorder. In addition, *TMPRSS6* was previously described as strongly associated with RBC volume and Hb concentration that affected MCV and MCH levels, respectively [7,9]. A genome-wide association study has mentioned that variation between the T and C alleles of SNP rs855791 could determine different RBC indices and iron status. The study reported that the T allele affected RBC volume and Hb concentration by decreasing MCV and MCH [7].

In this study, three SNPs genotypes, including C/C, T/C, and T/T, were found in the studied Thai population. The allele frequency of SNP rs855791 of the T to C allele is 0.6 to 0.4, which corresponds to allele frequency in the Asian population [9,11]. 

This study firstly determined the effects of SNP rs855791 (V736A) variation in variable RBC index parameters in the Thai population based on different α-globin genotypes, which were found in nearly 30% of the Thai population. The studied populations included three groups depending on α-globin genotypes, including αα/αα, -α/αα, and --/αα. Commonly, numbers of deleted alleles of the α-globin gene could decrease levels of MCV and MCH in α-thal carriers [14,15]. The results showed that SNP variation could determine levels of MCV and MCH with an increase in the C allele in the population carrying deletional α-globin genotypes. This suggests that the C allele of SNP rs855791 responded to deletion of the α-globin gene for maintenance of the RBC volume and Hb levels in RBCs to prevent the microcytic hypochromic anaemia condition in this population. 

This study also determined the effects of SNP rs855791 on iron status in the population with normal α-globin genotype and α-thal carriers. The population of IDA condition provided by WHO criteria [19] was excluded to prevent interference by iron deficiency condition. The study showed that the C allele could increase serum ferritin levels compared with the T allele in α^+^-thal and α^o^-thal, but the increasing levels did not achieve a statistical difference. Hepcidin levels and other iron parameters were not measured in this study. Therefore, the association between SNP rs855791 and hepcidin levels could not be analysed. However, a previous study described that the C allele could inhibit hepcidin more efficiently than that of the T allele, leading to iron parameters being higher in individuals carrying C alleles, especially C/C, than in T alleles [8]. These results may be interpreted as SNP rs855791 did not respond strongly to deletional mutations of the α-globin gene for iron regulation. 

## 5. Conclusions

The study discovered that a common SNP rs855791 of *TMPRSS6* associated with α-globin deletion played a role as a genetic factor affecting the RBC indices. This would be helpful for understanding the factor to maintain RBC indices in α-thal carriers, which have a high prevalence in the Thai population. The genetic mechanism involved in the determination of RBC indices in α-thal mutations should be further studied.

## Figures and Tables

**Table 1:** Demographic data of participant subjects of this study.

1	Total number of cases (N)	433	
2	Age (year, mean ± SD)	23.0 ± 8.7	
3	Sex: N (%)		
	3.1 Male	141	(32.56%)
	3.2 Female	292	(67.44%)
4	Globin genotype: N (%)		
	4.1 αα/αα, β/β		
	-Male	118	(27.25%)
	-Female	239	(55.20%)
	4.2 -α/αα, β/β		
	-Male	20	(4.62%)
	-Female	41	(9.47%)
	4.3 --/αα, β/β		
	-Male	3	(0.69%)
	-Female	12	(2.77%)

**Table 2:** Genotypic prevalence and allele frequency of SNP rs855791 of *TMPRSS6*.

Parameter	SNP	α-Globin Genotype					
	V736A	αα/αα		-α/αα		--/αα	
Genotypic	C/C						
prevalence:	Male	7	(3.93%)	3	(0.69%)	1	(0.23%)
N (%)	Female	33	(7.62%)	5	(1.15%)	3	(0.69%)
	T/C						
	Male	56	(12.93%)	12	(2.77%)	0	(0.00%)
	Female	114	(26.33%)	26	(6.00%)	5	(1.15%)
	T/T						
	Male	45	(10.39%)	5	(1.15%)	2	(0.46%)
	Female	92	(21.25%)	10	(2.31%)	4	(0.92%)
Allele	C						
frequency	Male	0.4		0.4		0.4	
	Female	0.4		0.4		0.4	
	T						
	Male	0.6		0.6		0.6	
	Female	0.6		0.6		0.6	

## Data Availability

Data of this study are available within the article.
